# Secular Trends in Soda Consumption, California, 2011–2016

**DOI:** 10.5888/pcd16.180372

**Published:** 2019-05-16

**Authors:** Matthew M. Lee, Jennifer Falbe, Kristine A. Madsen

**Affiliations:** 1 University of California, Berkeley, Community Health Sciences, School of Public Health, Berkeley, California; 2 University of California, Davis, Human Development and Family Studies, Department of Human Ecology, Davis, California

## Abstract

Consumption of sugar-sweetened carbonated beverages (including soda) has declined nationally, but trends by state are unknown. We used data from the California Health Interview Survey to assess overall changes in soda consumption among adults aged 18 or older from 2011 through 2016 and identified differences by education and income level. Frequency of soda consumption (times per week) declined from 2011 through 2014 by 16.5% but returned to 2011 levels in 2015 and 2016; trends did not differ by education or income. The proportion of the population that consumed soda did not change among adults with less than a high school diploma or equivalent, but declined significantly among those with at least a high school diploma. Our findings suggest that soda consumption remains a pressing public health problem in California.

**Summary**

**What is already known about this topic?**

Consumption of sugar-sweetened beverages (SSBs) has declined nationally, but trends by state are unknown. In California, Albany, Berkeley, Oakland, and San Francisco have passed taxes on SSBs.

**What is added by this report?**

We highlight soda consumption trends at the California state level. While consumption frequency declined from 2011 through 2014, levels in 2015–2016 were similar to 2011 levels. The proportion of soda consumers did not change for those without a high school diploma, but declined for those with at least a high school diploma.

**What are the implications for public health practice?**

By understanding state soda consumption trends, researchers and policymakers can monitor and contextualize consumption changes at the city-level following taxation of SSBs.

## Objective

Consumption of sugar-sweetened beverages (SSBs), such as sodas, is associated with increased risk of diabetes and cardiovascular disease (1) and is inversely correlated with income and education. Healthy People 2020 objectives include reducing calories from added sugars (2), and numerous local jurisdictions have proposed or adopted taxes aimed at curbing SSB consumption, including 4 California cities (Albany, Berkeley, Oakland, San Francisco) (3–6). Monitoring state-level consumption patterns provides context and comparison for city-level interventions. Hence, our objective was to use a population-based sample to estimate trends in soda consumption (the SSB with highest consumption levels) (7) in California from 2011 through 2016, considering income and education as effect modifiers.

## Methods

We used data from the California Health Interview Survey (CHIS) to estimate weekly frequency of soda consumption from 2011 through 2016 among adults aged 18 or older. CHIS is an annual population-based telephone survey that uses complex multistage sampling and survey weights (currently based on 2010 Census projections) to provide estimates that are generalizable to California’s overall population (8). CHIS response rates ranged from 42% to 47% for the landline respondent sample and from 48% to 54% for cellular telephone respondents. Detailed information on the CHIS sampling methodology is available elsewhere (8). This research was considered exempt from review by the University of California, Berkeley, Committee for the Protection of Human Subjects.

Among other demographic and health-related information, CHIS participants were asked to report their soda consumption by responding to the question, “During the past month, how often did you drink regular soda or pop that contains sugar? Do not include diet soda.” All responses were converted to frequency of consumption per week. Data from 2011 through 2016 were pooled by following CHIS guidelines (9). By using a generalized linear model with a log link and γ distribution (because soda consumption is nonnegative and right skewed), we estimated the ratio of mean weekly soda consumption each year from 2012 through 2016 relative to consumption in 2011. We adjusted for sex, age, language, race, income as a percentage of the federal poverty level (FPL) (0%–99%, 100%–199%, 200%–299%, or ≥300%), and education (less than high school diploma, high school diploma or general equivalency diploma, some college, college degree or more), and we explored interaction separately by income (<200% of FPL) and education (less than a high school diploma). To obtain inference for the population in California, we applied survey weights and used jackknife replication to construct standard errors and 95% confidence intervals. We also implemented a Poisson regression model with robust standard errors (SEs) to estimate the proportion of the California adult population that consumed soda in each year. Analyses were conducted in Stata/SE 15.1 (StataCorp LLC).

## Results

By applying survey weights to the CHIS sample, we examined differences that were directly attributable to changes in the actual California adult population and not to yearly sampling differences in CHIS. Demographics differed by year, and 2-tailed χ^2^ tests for each covariate returned *P* values of <.001. The distribution of covariates over time was heterogeneous and nonmonotonic, suggesting that adjustment by multivariate regression was necessary. The proportion of white participants was 45.6% in 2011, but 41.8% in 2016, coinciding with an overall 8.1% increase in California’s adult population, from 27.2 to 29.4 million (Table).

**Table:** Population-Weighted Descriptive Characteristics of California Residential Noninstitutionalized Adults Aged 18 or Older, 2011–2016^a^

Characteristic	2011 (n = 22,580)^b^	2012.(n = 20,355)^b^	2013 (n = 20,724)^b^	2014 (n = 19,516)^b^	2015 (n = 21,034)^b^	2016 (n = 21,055)^b^
**Total population, millions**	27.2	27.8	28.2	28.5	29.1	29.4
**Sex**						
Male	13.3 (48.9)	13.5 (48.7)	13.7 (48.7)	13.9 (48.9)	14.2 (48.9)	14.4 (48.9)
Female	13.9 (51.1)	14.3 (51.3)	14.5 (51.3)	14.6 (51.2)	14.9 (51.1)	15.0 (51.1)
**Age, y**						
18–29	6.4 (23.6)	6.5 (23.5)	6.6 (23.3)	6.5 (22.9)	6.4 (22.1)	6.4 (21.8)
30–39	4.7 (17.3)	5.0 (18.1)	5.1 (18.1)	5.2 (18.1)	5.3 (18.2)	5.4 (18.2)
40–49	5.3 (19.4)	5.1 (18.3)	5.0 (17.9)	5.0 (17.6)	5.1 (17.5)	5.1 (17.3)
50–59	4.8 (17.5)	4.8 (17.4)	4.8 (17.0)	5.0 (17.6)	5.0 (17.2)	4.7 (15.9)
≥60	6.1 (22.3)	6.3 (22.7)	6.7 (23.7)	6.8 (23.8)	7.3 (25.1)	7.9 (26.8)
**Language**						
English	23.5 (86.5)	23.1 (83.1)	24.2 (85.8)	24.0 (84.1)	25.5 (87.8)	24.7 (83.9)
Spanish	3.7 (13.5)	4.7 (16.9)	4.0 (14.2)	4.5 (15.9)	3.6 (12.2)	4.7 (16.1)
**Race/ethnicity**						
African American	1.5 (5.6)	1.6 (5.7)	1.6 (5.6)	1.6 (5.7)	1.6 (5.6)	1.6 (5.6)
White	12.4 (45.6)	12.1 (43.6)	12.2 (43.1)	12.1 (42.6)	12.3 (42.2)	12.3 (41.8)
Asian, Native Alaskan, mixed	4.2 (15.4)	4.5 (16.3)	4.6 (16.4)	4.7 (16.6)	4.9 (16.7)	4.9 (16.8)
Hispanic	9.1 (33.4)	9.6 (34.4)	9.8 (34.9)	10.0 (35.1)	10.3 (35.4)	10.5 (35.7)
**Education**						
<High school diploma	4.3 (15.9)	4.4 (16.0)	4.4 (15.5)	4.3 (15.1)	5.0 (17.3)	5.0 (17.0)
High school diploma or GED	6.6 (24.3)	6.7 (24.2)	6.9 (24.4)	6.9 (24.3)	6.4 (21.9)	6.5 (22.0)
Some college	6.6 (24.4)	7.0 (25.3)	7.3 (26.0)	7.4 (25.8)	7.0 (24.0)	6.9 (23.3)
≥College degree	9.7 (35.5)	9.6 (34.4)	9.6 (34.2)	9.9 (34.8)	10.7 (36.9)	11.1 (37.7)

Abbreviation: GED, general equivalency diploma.

a Values are number in millions (percentage) unless otherwise indicated.

b Numbers are from the California Health Interview Survey (8).

Across all years, adults with incomes less than 200% of FPL and those with less than a high school diploma or equivalent consumed more soda than adults with higher incomes or education (*P* < .001). Compared with 2011, adjusted mean frequency of soda consumption was 7.6% lower in 2012 (95% confidence interval [CI], 1.2%–13.7%), 11.3% lower in 2013 (95% CI, 4.4%–17.7%), 16.5% lower in 2014 (95% CI, 8.3%–24.0%), but not significantly different in 2015 or 2016 (Box); neither race nor income were significant effect modifiers. On average, California adults consumed 2.1 sodas per week in 2011, 1.9 in 2012, 1.9 in 2013, 1.8 in 2014, 2.1 in 2015, and 2.0 in 2016. Among adults with a high school diploma or greater, the adjusted proportion consuming any soda in the previous month was 44.0% in 2011, with significantly lower proportions (*P* < .001 for 2013 and 2014, *P* *=* .008 for 2015, *P* = .014 for 2016) in all subsequent years except 2012 (at the nadir in 2014, 37.3% consumed soda in the previous week) (Figure). In contrast, in 2011 among adults with less than a high school diploma, 47.0% reported consuming soda in the previous week, with no differences in the proportion consuming soda in subsequent years compared with 2011. Income was not a significant effect modifier.

**Box. Average Weekly Frequency of Soda Consumption Among California Adults Aged 18 or Older, Adjusted for Education Level, Race/Ethnicity, Sex, Age, Language, and Annual Income as a Percentage of the Federal Poverty Level, 2011–2016**

**Table:** 

Year	Estimated Weekly Frequency, Times/Week (95% Confidence Interval)
2011	2.10466 (2.00687–2.20245)
2012	1.94384 (1.83962–2.04807)
2013	1.86707 (1.75049–1.98365)
2014	1.75635 (1.60756–1.90513)
2015	2.10366 (1.90713–2.30019)
2016	2.04671 (1.85087–2.24256)

**Figure:**
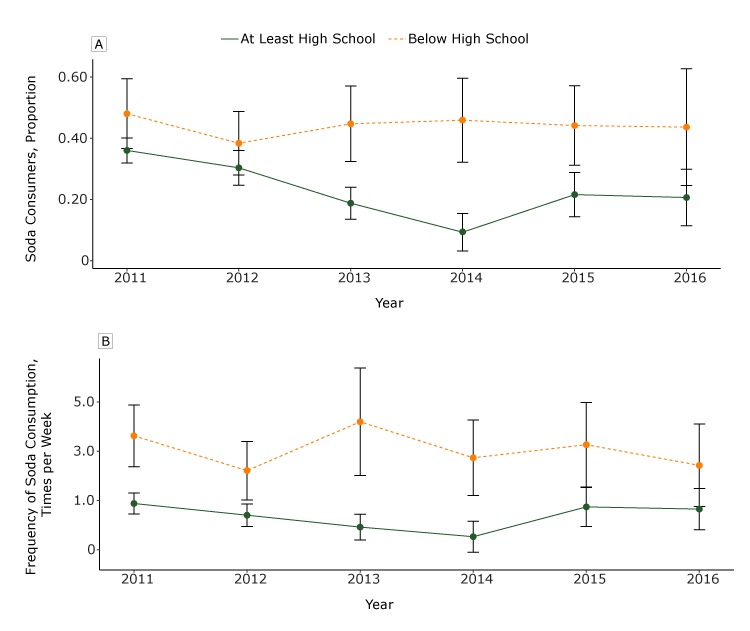
Adjusted weekly soda consumption (excluding diet soda) and proportion of California adults aged 18 or older who consumed soda, from 2011 through 2016. A. Adjusted proportion of California adults who consumed any soda in the previous week, by year and education level (at least a high school diploma or equivalent compared with less than a high school diploma). Among those with a high school diploma or above, there were significant within-group differences (from 2011) in 2013 and 2014 (*P* < .001) and 2015 and 2016 (*P* < .01), and in 2014 there were significant between-group differences (*P* = .004). B. Adjusted estimated mean frequencies of weekly soda consumption, by year and education status (at least a high school diploma or equivalent compared with less than a high school diploma). Among those with a high school diploma or above, there were significant differences (from 2011) in weekly consumption frequency in 2013 (*p* = 0.004) and 2014 (*P* = .001). Consumption frequency was modeled by using a log link and gamma distribution, whereas the proportion of the California adult population who were soda consumers was modeled by using a log link and Poisson distribution with robust standard errors. All analyses were adjusted for education, race/ethnicity, sex, age, language, and income as a percentage of federal poverty level. Brackets indicate confidence intervals.

**Table:** 

Year	Less Than High School Diploma or Equivalent	High School Diploma or More
**A. Soda consumers, proportion (95% Confidence Interval)**		
2011	47.0 (44.2-49.9)	44.0 (43.0–45.0)
2012	44.6 (42.0–47.2)	42.6 (41.2–44.0)
2013	46.2 (43.1–49.3)	39.7 (38.4–41.0)
2014	46.5 (43.0–49.9)	37.3 (35.8–38.9)
2015	46.0 (42.8–49.3)	40.4 (38.6–42.2)
2016	45.9 (41.1–50.7)	40.2 (37.9–42.5)
**B. Soda consumption, frequency in times/week (95% Confidence Interval)**		
2011	2.66 (2.34–2.97)	1.97 (1.86–2.08)
2012	2.30 (2.01–2.60)	1.85 (1.74–1.96)
2013	2.80 (2.25–3.34)	1.73 (1.60–1.86)
2014	2.43 (2.05–2.82)	1.63 (1.47–1.79)
2015	2.57 (2.14–2.99)	1.93 (1.74–2.13)
2016	2.36 (1.94–2.77)	1.91 (1.70–2.12)

## Discussion

The decreased consumption of soda in California from 2012 through 2014 relative to 201l is consistent with national declines reflected in data from the National Health and Nutrition Examination Survey (10) and the Behavioral Risk Factor Surveillance System (11). National data from 2014 and forward have not yet been published, and trends in soda consumption could mirror the rebound seen in California after 2014, which would be a setback for public health. Although 2014 was the year Berkeley passed the first SSB tax in the United States, the nadir in consumption we report in 2014 is unlikely to be related to associated media attention: 3 additional California cities passed taxes in 2016 with no state-wide decline in consumption.

Our study demonstrated differential changes in consumption of any soda based on education level, which is a potential source of health inequity. Excess soda consumption has serious health consequences for the more than 3 million California adults with less than a high school diploma. Given the lack of healthy, affordable food options and the use of targeted marketing of “junk food” in low-income communities, interventions are needed that reduce the gap in soda consumption and reduce, rather than perpetuate, health disparities (12).

Our findings are timely given ongoing evaluations of SSB taxes in California and the recent pre-emption of any new local SSB taxes in California (pre-emption prohibits additional local jurisdictions from passing their own SSB taxes within the next 12 years, but taxes in cities that have already implemented them [Albany, Berkeley, San Francisco, and Oakland] remain in effect) (13). Taxes on SSBs have been enacted at the city level but not at the state or federal level, and initial reports on the effect of these taxes suggest that they reduce consumption locally (14,15). Future studies should assess long-term effects on consumption and population health (16).

Our study had limitations. CHIS data are self-reported and susceptible to recall and social-desirability biases, and CHIS did not assess other SSBs such as sports, energy, and fruit-flavored drinks. Unmeasured confounding is a concern in any observational study. Although variability in CHIS methods between cycles may have increased bias if changes modified exposure–outcome assessment, consistency in questionnaires between years and extensive measures to minimize nonresponse make this bias unlikely in our study (17).

Soda consumption in California remains a pressing public health problem, and consumption in 2016 was no different than in 2011. Continued surveillance and interventions that support low-income communities in decreasing consumption are needed to reduce diet-related chronic illnesses.
